# Contraceptive Method Choices in Women With and Without Opioid Use Who Have Infants in the Neonatal Intensive Care Unit and Nursery

**DOI:** 10.1089/whr.2019.0025

**Published:** 2020-09-24

**Authors:** Alia Radwan, Bobbie Nicole Ray, David M. Haas

**Affiliations:** Department of Obstetrics and Gynecology, Indiana University School of Medicine, Indianapolis, Indiana, USA.

**Keywords:** opioid use disorder, postpartum contraception, immediate contraception

## Abstract

***Objective:*** The aim of this study was to examine whether a history of opioid use predicts tier 1 contraceptive use or plan to use in women with infants in the neonatal intensive care unit (NICU) and nursery.

***Materials and Methods:*** We conducted a self-administered, anonymous survey in women with infants in three local NICUs and two postpartum units from November 2018 to May 2019. Women were recruited while visiting their infants in the NICU or in their postpartum rooms. Our survey included adapted questions from the Centers for Disease Control and Prevention (CDC) Pregnancy Risk Assessment Monitoring System (PRAMS) questionnaire, the National Institute of Drug Abuse (NIDA) Modified ASSIST Screening Tool, and ones written by our team. The questions asked about contraceptive use and opioid use. We compared the responses of women with and without a history of opioid use. We conducted a multivariable regression analysis and applied the backward elimination method to identify whether opioid use was a predictor of tier 1 contraceptive use or plan to use.

***Results:*** A total of 122 women completed the survey. Fifty-three women (43.4%) reported opioid use in the month before pregnancy and/or during pregnancy, while 69 (56.6%) women reported no opioid use and comprised the control group. Multivariable regression analysis showed that opioid use was not associated with the use or planned use of tier 1 contraceptives (adjusted odds ratio [aOR] 1.47; confidence interval [95% CI] 0.54–4.01). Older maternal age predicted tier 1 choice (aOR 1.12; 95% CI 1.04–1.21), while African American women were less likely to use or plan to use tier 1 contraceptives compared with white women (aOR 0.21; 95% CI 0.08–0.56).

***Conclusion:*** A history of opioid use was not independently associated with women using or planning to use tier 1 methods, while age and race were predictors.

## Introduction

Women who use opioids are at high risk for unintended pregnancy, with rates ranging from 75% to 86%, compared with 51% in the general population.^1–3^ The Centers for Disease Control and Prevention (CDC) recently reported that the number of American women with opioid use disorder (OUD) admitted to labor and delivery has quadrupled from 1999 to 2014.^4^ This increase is reflected in the higher prevalence of neonatal opioid withdrawal syndrome (NOWS) (also referred to as neonatal abstinence syndrome [NAS]) in the United States.^5,6^ Opioid abuse in pregnancy is also associated with adverse fetal, neonatal, and maternal outcomes such as preterm birth, intrauterine growth restriction, and long-term developmental delays.^7–9^

In the context of the high unintended pregnancy rate, particularly among women who use substances, understanding factors related to contraceptive use can inform providers and guide public health efforts to reduce unintended pregnancies. Our study sought to better characterize contraceptive choices among postpartum women who used opioids because of the growing opioid epidemic in Indiana.^10^ The early postpartum time frame is an opportunity for women to develop plans for birth spacing. We also wanted to learn about the contraceptive choices of women with infants in the neonatal intensive care unit (NICU) since low use of effective contraception in women with preterm infants puts them at high risk for unintended pregnancy and recurrent preterm birth.^11,12^ To our knowledge, there are few studies looking specifically at postpartum contraceptive choices of these women.

The objective of our study was to determine postpartum contraception choices among women with infants in the NICU and nursery, comparing rates of tier 1 contraceptive^13,14^ use or plan to use in women who did and did not use opioids.

## Materials and Methods

### Recruitment

This cross-sectional, anonymous survey was conducted in women with infants in the NICU and nursery in two main delivery hospitals and a local children's hospital in Indianapolis, Indiana, between November 2018 and May 2019. The study was approved by the Indiana University Institutional Review Board and Eskenazi Health Research Committee.

We recruited women with and without a history of opioid use who had infants in the NICU. We also recruited women with a history of opioid use whose infants did not meet the criteria for NICU admission and therefore remained in the room with them or in the nursery as they were being observed for NOWS. Other inclusion criteria included that the participants were at least 18 years old and English speaking. Potential participants were identified through chart screening and input from the nursing staff. Exclusion criteria included mothers who were incarcerated or under house arrest. We coordinated with the NICU and postpartum nursing units to exclude women with mental distress. The first author, accompanied by a research assistant, approached potential participants either while the women were visiting their infants in the NICU or in their postpartum rooms. At first encounter with the women, the first author explained the goals of the study and willing participants gave consent by completion of the survey. Our self-administered survey was conducted on a department laptop using REDCap.^15,16^

At initiation of the study, both groups (women with and without a history of opioid use) were recruited and the numbers were monitored periodically to achieve similar sample sizes. When there was a notable difference in the sample sizes of the two groups, we stopped recruiting the control group and continued to recruit opioid using women to attain more even sample sizes.

### Survey procedures—contraceptive methods

To examine which contraceptive methods our participants selected, we asked “which birth control method have you received or are you planning to use or receive now or in the next month?” The question and list of options were adapted from the CDC Pregnancy Risk Assessment Monitoring System (PRAMS) questionnaire.^17^ Women who chose female sterilization or a long-acting reversible contraceptive (LARC) method answered a follow-up question about when they received or planned to receive this method in order for us to ascertain whether the timing of the tier 1 method use was immediate or a future intent.

Participants who selected sterilization or a LARC method were coded in the tier 1 category. Those who chose a tier 2 method such as oral contraceptive pills, transdermal patch, vaginal ring, or depo medroxyprogesterone injections, were coded as tier 2. We coded women who selected condoms, natural family planning, or withdrawal method from our list options into a category termed “lower tiers.” The one participant who selected “other” was coded as such. Women who answered “abstinence” or “none” were coded as “none.” For participants who selected multiple contraceptive methods of different tiers, the higher (more effective) tier method was recorded.

### Opioid use

**FIG. 1.**
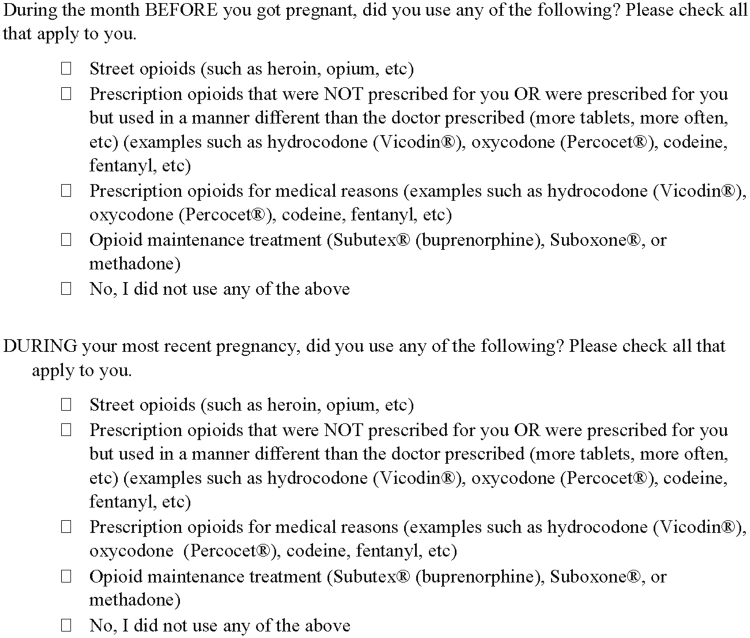
Opioid use questions. Note the question stem is adapted and slightly modified from the CDC PRAMS questionnaire.^17^ The answer choices are from the CDC PRAMS questionnaire,^17^ NIDA Modified ASSIST Screening Tool,^18^ and by us. CDC, Centers for Disease Control and Prevention; NIDA, National Institute of Drug Abuse; PRAMS, Pregnancy Risk Assessment Monitoring System.

With regard to inquiring about opioid use, we adapted questions and answers from the PRAMS questionnaire^17^ and the National Institute of Drug Abuse (NIDA) Modified ASSIST Screening Tool.^18^ Figure 1 displays the modified questions we used in our survey. Women who report use of street opioids and/or opioid maintenance therapy (OMT) were included in the opioid using group. We also included women who reported use of any opioid prescriptions before and/or during pregnancy in the opioid using group since opioid prescriptions are prevalent in pregnant women.^19^

### Maternal characteristics

The survey additionally gathered demographic and background information from the participants regarding their age, race/ethnicity, relationship status, education and income levels, type of insurance, parity, mode of delivery, medical conditions, due date, delivery date, and whether their recent delivery was preterm. Gestational age at birth was calculated from participant reported due date and delivery date. Additional questions about pregnancy intentions and attitudes were also asked.^20,21^

### Analysis

Maternal characteristics and contraceptive outcomes were compared using independent samples *t*-test for continuous variables and the chi-square test for categorical variables. We also performed a bivariate analysis to compare opioid use and clinically relevant variables (mean maternal age, mean gestational age, race/ethnicity, parity, preterm birth, education level, and stable partner) between those who planned to use or are already using tier 1 contraceptives versus not. We then conducted a multivariable logistic regression analysis while controlling for the potential confounders. In our final model, we applied the backward elimination method in our multivariable regression analysis set at 0.1 that included all of the above variables. Multicollinearity was not found when we examined variation inflation factors. Adjusted odds ratios (OR) and confidence intervals (95% CI) were calculated. Data analysis was conducted with the SPSS 25 software.

## Results

### Characteristics

We approached an estimated 145 women, of whom 125 participated (86% response rate). Three women were excluded from the study due to their incomplete responses. The women who declined participation (*n* = 20) indicated that it was not a convenient time for them since they were caring for their infants.

Of the 122 women who completed the survey, 53 (43.4%) reported opioid use in the month before pregnancy and/or during pregnancy and 69 (56.6%) denied use and were categorized as controls. Of the 53 women in the opioid use category, 49 (92.5%) reported OMT use, 7 women reported intake of prescriptions that were not prescribed for them or took incorrectly (of which 6 also noted OMT use), and 7 noted use of prescription opioids for medical reasons (of which 5 noted OMT use).

The characteristics of the women who completed the survey are described in Table 1. Women who reported opioid use tend to be older, white, have a higher parity, have a lower education, and lower income.

**Table 1.** Characteristics of Participants (*n* = 122)

Characteristics	Control (n = 69)	Opioid (n = 53)	p^a^
Mean maternal age (years)	27.2 ± 6.0	29.8 ± 5.1	0.015
Mean gestational age (weeks)	33.9 ± 5.0	37 ± 3.4	<0.001
Race/ethnicity			
White	36 (52.2)	47 (88.7)	<0.001
African American	22 (31.9)	5 (9.4)	
Hispanic	7 (10.1)	0	
Other	4 (5.8)	1 (1.9)	
Education			
High school or less	34 (49.3)	38 (71.7)	0.016
Some college or more	34 (49.3)	15 (28.3)	
Did not answer	1 (1.4)	0	
Relationship status			
Single	15 (21.7)	18 (34)	0.151
Stable partner	54 (78.3)	34 (64.1)	
Did not answer	0	1 (1.9)	
Income per year			
Less than $30,000	26 (37.7)	34 (64.1)	0.012
$30,000–$49,999	6 (8.7)	7 (13.2)	
>$50,000	15 (21.7)	1 (1.9)	
I do not wish to answer/left blank	22 (31.9)	11 (20.8)	
Type of insurance			
Medicaid	40 (58)	47 (88.7)	0.001
Private	15 (21.7)	5 (9.4)	
Other	10 (14.5)	0	
None	4 (5.8)	1 (1.9)	
Parity			
1	30 (43.5)	11 (20.8)	0.006
2	13 (18.8)	9 (17)	
3	17 (24.6)	13 (24.5)	
4 or more	9 (13)	20 (37.7)	
Opioid maintenance therapy (*N* = 47)			
Subutex^®^ (buprenorphine)	N/A	27 (57.4)	
Suboxone^®^	N/A	7 (14.9)	
Methadone	N/A	13 (27.7)	
Preterm birth (current pregnancy)	41 (59.4)	13 (24.5)	<0.001
Mode of delivery			
Vaginal	36 (52.2)	31 (58.5)	0.564
Cesarean delivery	32 (46.4)	22 (41.5)	
Both (twins)	1 (1.4)	0	
Desires future pregnancy in			
1–2 years	6 (8.7)	0	0.003
2 or more years	20 (29)	5 (9.4)	
Unsure	15 (21.7)	19 (35.8)	
I do not want any more	28 (40.6)	29 (54.7)	
I do not know	2 (2.9)	0 (0)	
Breastfeeding			
No	19 (27.5)	29 (54.7)	0.002
Yes	50 (72.5)	24 (45.3)	
Conditions			
Hypertensive disease in pregnancy	9 (13)	10 (18.9)	0.262
Gestational diabetes	10 (14.5)	0	
PTL/PPROM	21 (30.4)	11 (20.7)	
Placental problem	9 (13)	4 (7.5)	
Blood clots	1 (1.4)	2 (3.8)	
Depression/anxiety	8 (11.6)	11 (20.7)	
Other	2 (2.9)	2 (3.8)	
Not diagnosed with any of the above	27 (39.1)	28 (52.8)	
Left blank	1 (1.4)	0	

Data are represented as *n* (%), unless noted as means in which case it is mean ± standard deviation.

^a^ *p*-Values were calculated through chi-square tests for categorical variables or *t*-tests for continuous variables.

PPROM, preterm prelabor rupture of membranes; PTL, preterm labor.

Regarding overall contraceptive selections, 76 women (62.3%) reported using or planning to use a tier 1 method, with 32 (42.1%) marking female sterilization and 42 (55.3%) marking use of an LARC (two women selected multiple answers). Twenty-nine (23.8%) reported use or plan to use tier 2, with oral contraceptives being the most endorsed method in this tier (*n* = 17). Lower tier methods were chosen by eight women, while nine endorsed no methods used or planned to use (Table 2).

**Table 2.** Contraceptive Outcomes, by Group (*n* = 122)

Contraceptive methods	Control (n = 69)	Opioid (n = 53)	p^a^
Plan to receive tier 1 contraceptive method before discharge or in the near future	23 (33.3)	9 (16.9)	0.04
Female sterilization	7 (10.1)	4 (7.5)	
LARC	16 (23.2)	4 (7.5)	
Reported multiple answers^b^	0	1 (1.9)	
Received tier 1 contraceptive method immediate postplacental	13 (18.8)	31 (58.5)	<0.001
Female sterilization	8 (11.6)	13 (24.5)	
LARC	5 (7.2)	17 (32.1)	
Reported multiple answers^c^	0	1 (1.9)	
Tier 2 contraceptive method	18 (26.1)	11 (20.8)	0.49
Oral contraceptives	10 (14.5)	7 (13.2)	
Injection	8 (11.6)	3 (5.7)	
Vaginal ring	0	1 (1.9)	
Transdermal patch	0	0	
Lower tiers^d^	7 (10.1)	0	0.02
Other	1 (1.4)	0	0.38
None	7 (10.1)	2 (3.8)	0.18

Data are represented as *n* (%).

^a^ *p*-Values were calculated through chi-square tests.

^b^ One patient selected two answer choices, tubal ligation and IUD.

^c^ One patient selected two answer choices, tubal ligation and IUD.

^d^ Lower tier includes tier 3 and tier 4 methods according to the WHO classification^13,14^: barrier contraceptives, natural family planning, withdrawal, other).

IUD, intrauterine device; LARC, long-acting reversible contraceptive.

Comparing contraceptive outcomes between the two groups, 40 (75.5%) in the opioid using group reported use or plan to use of a tier 1 contraceptive method versus 36 (52.2%) in the control group (Table 2). Tier 2 methods represented the second largest composition of contraceptives with 20.8% of opioid using women and 26.1% of the control group opting for this tier. None in the opioid use group selected lower tier methods versus 10.1% in the control group. Small percentages of either group opted for “none” (Table 2).

Exploring the timing of tier 1 contraceptive initiation, 31 (58.5%) in the opioid using group opted for immediate postpartum contraception uptake in comparison with 13 women (18.8%) in the control group (*p* < 0.001) (Table 2). Women who used opioids were more likely to use or planned to use a tier 1 method, as were women who were older, white, and had higher parity (Table 3).

**Table 3.** Characteristics of Women Who Are Using or Planning to Use a Tier 1 Contraceptive Method (*n* = 122)

Variable	Using or planning to use tier 1 contraceptive^a^		
	No	Yes	p^b^
	46	76	
Opioid use	13 (28.3)	40 (52.6)	0.008
Mean maternal age (years)	26.3 ± 5.9	29.6 ± 5.3	0.002
Mean gestational age (weeks)	34.8 ± 5.0	35.6 ± 4.8	0.34
Race/ethnicity			
White	24 (52.2)	59 (77.6)	0.017
African American	17 (36.9)	10 (13.2)	
Hispanic	3 (6.5)	4 (5.3)	
Other	2 (4.3)	3 (3.9)	
Parity			
1	23 (50)	18 (23.7)	0.008
2	9 (19.6)	13 (17.1)	
3	9 (19.6)	21 (27.6)	
4 or more	5 (10.9)	24 (31.6)	
Preterm birth	22 (47.8)	32 (42.1)	0.62
Some college education	20 (43.5)	29 (38.2)	0.49
Stable partner	32 (69.6)	56 (73.7)	0.54

Data are represented as *n* (%), unless noted as means in which case it is mean ± standard deviation.

^a^ Tier 1 contraceptive methods according to the WHO classification^13,14^: female/male sterilization, intrauterine device, and contraceptive implant.

^b^ *p*-Values were calculated through chi-square tests for categorical variables or *t*-tests for continuous variables.

In a multivariable regression analysis (Table 4), opioid use was not independently associated with tier 1 method use or plan to use (OR 1.47; 95% CI 0.54–4.01). We found that after performing a backward elimination regression model, the African American race (OR 0.21; 95% CI 0.08–0.56) remained predictive of lower tier 1 method use or plan to use, while older maternal age (OR 1.12; 95% CI 1.04–1.21) was predictive of higher odds to use or plan to use a tier 1 contraceptive (Table 4).

**Table 4.** Predictors of Tier 1 Contraceptive Use or Plan to Use (*n* = 122)

Variable	Multivariable regression analysis adjusted OR (95% CI)	Backward regression analysis adjusted OR (95% CI)
Opioid use	1.47 (0.54–4.01)	
Maternal age	1.08 (0.97–1.19)	1.12 (1.04–1.21)
Gestational age	0.95 (0.82–1.11)	
Race/ethnicity		
White	Reference	Reference
African American	0.17 (0.05–0.58)	0.21 (0.08–0.56)
Hispanic	0.80 (0.13–4.88)	0.70 (0.14–3.61)
Other	0.68 (0.09–5.40)	0.50 (0.07–3.32)
Parity		
1	Reference	
2	1.47 (0.41–5.24)	
3	2.33 (0.66–8.23)	
4 or more	3.53 (0.81–15.32)	
Preterm birth	1.51 (0.58–3.95)	
Some college education	0.80 (0.29–2.17)	
Stable partner	1.46 (0.54–3.96)	

CI, confidence interval; OR, odds ratio.

## Discussion

Our study provides insight on correlates of highly effective contraceptive plans among women who are at risk for an unintended pregnancy. When adjusting for other relevant covariates, opioid use in our study was not associated with tier 1 method use or plan to use. This was consistent with a systemic review that reported approximately half of women with substance use utilized contraception, of which only 8% used a LARC.^22^ This is in contrast to 14% LARC use rates reported in the general population.^23^ Low uptake of contraception in opioid using women has been attributed to numerous factors, including poor postpartum visit attendance,^3,24,25^ stigma and fear of losing custody of their child(ren),^26^ lack of information regarding both LARCs and short-acting methods,^27^ and misunderstanding of their fertility.^3,28^

Interestingly, we also found that more than half of women with self-reported opioid use in our study chose immediate tier 1 method uptake, of which 55% selected an LARC method. This suggests that immediate postpartum LARC insertion is a preferred option for opioid using women in our population who choose long-term methods. To our knowledge, immediate contraceptive uptake in women who use opioids has not been well examined, but studies in general postpartum populations have demonstrated that LARC users desire immediate initiation.^29^ In fact, one institution that did not previously offer immediate postpartum LARC insertion noted that while there is interest and intent in LARC use in women with OUD, many women did not receive them in the interval period.^24^

We also found that older women more often chose tier 1 contraceptive methods. This is likely due to the choice of sterilization in women who have satisfied parity.^30^ In addition, we found that African American women were less likely to choose a tier 1 method. In our system, the majority of women were on Medicaid and the cost of a tier 1 method would typically not be a limiting factor for choosing one of these tier 1 methods. Given higher rates in preterm birth and maternal morbidity and mortality in African American women,^31,32^ addressing this disparity in use of effective contraception is an area that can be focused on in future work.

Offering immediate effective contraception may reduce some of the barriers that hinder women from receiving an LARC method.^33^ However, there are concerns about force or coercion that raise ethical questions about influencing the contraceptive choices in women with substance use disorders.^34,35^ The role of the provider as a potential contributor to health disparities in disadvantaged groups^36,37^ is a sensitive area that may affect various vulnerable populations. Therefore, further studies should elucidate the role of provider influence in encouraging immediate contraceptive uptake in women who use substances.

The contraceptive choices of the control group are also notable in this study. One-third of the NICU nonopioid using mothers in this study were planning tier 1 method use, perhaps waiting on their infant's health status before committing to a long-term contraceptive. Previous studies in mothers with premature neonates in the NICU showed that a small percentage of women were aware of the major risk factors for recurrent preterm birth and had a lower uptake of effective contraception.^11,12^ Another study reported disparities in postpartum contraceptive counseling and effective contraceptive uptake in women with preterm infants.^38^ Since this is a high-risk group for repeat preterm deliveries, there should be a focus on their reproductive plans and perceptions about contraception.

Our study has the typical limitations and biases common in survey projects. Due to the anonymity of the survey, we did not follow the women to confirm receipt of tier 1 contraception. In addition, this study collected self-reported information and was not verified with a chart review. Also, there may be social desirability bias, hence underreporting of opioid use in patients. While there are a substantial number of women on OMT at one of our sites, some of them participate in group prenatal care and hence receive support and resources, and so, the results may not be representative of opioid using women who have limited or no prenatal care. Another limitation is that we did not ask about lactational amenorrhea (tier 2), diaphragm or sponge (tier 3), or spermicide (tier 4) since these methods tend to be infrequently used as primary methods in our population. A further limitation is that we did not ask about other substance or tobacco use as we solely focused on the opioid category.

## Conclusion

Promising outcomes in women's reproductive health have emerged from state initiatives and institutional programs that promote LARC use and increased access to family planning services.^39–41^ Hence, we would propose multiple strategies to increase LARC uptake in women who use opioids. In agreement with other authors, postpartum contraception counseling should be incorporated in routine prenatal visits as it is an important aspect in prenatal care.^42^ Since women with opioid use face barriers in prenatal care and care outside pregnancy, providers should consider incorporating women's health programs at treatment centers to increase education about contraceptive methods and dispel misinformation and common misperceptions about fertility. In addition, further investigations should seek to learn the approaches and language used by health care professionals when counseling and educating women who use opioids and other substances to eliminate patient perceptions of bias and judgment. Finally, as the opioid epidemic persists, efforts should be directed toward providing contraceptive access, particularly immediate while still inpatient, for the women who desire it.

## Abbreviations Used

CDC: Centers for Disease Control and Prevention
CI: confidence interval
LARC: long-acting reversible contraceptive
NAS: neonatal abstinence syndrome
NICU: neonatal intensive care unit
NIDA: National Institute of Drug Abuse
NOWS: neonatal opioid withdrawal syndrome
OMT: opioid maintenance therapy
OR: odds ratio
OUD: opioid use disorder
PRAMS: Pregnancy Risk Assessment Monitoring System

## References

[1] Heil SH, Jones HE, Arria A, et al. Unintended pregnancy in opioid-abusing women. J Subst Abuse Treat 2011;40:199–202 21036512 10.1016/j.jsat.2010.08.011 PMC3052960

[2] Finer LB, Henshaw SK Disparities in rates of unintended pregnancy in the United States, 1994 and 2001. Perspect Sex Reprod Health 2006;38:90–96 16772190 10.1363/psrh.38.090.06

[3] Fischbein RL, Lanese BG, Falletta L, Hamilton K, King JA, Kenne DR Pregnant or recently pregnant opioid users: Contraception decisions, perceptions and preferences. Contracept Reprod Med 2018;3:4 29610676 10.1186/s40834-018-0056-y PMC5870942

[4] Haight SC, Ko JY, Tong VT, Bohm MK, Callaghan WM Opioid use disorder documented at delivery hospitalization—United States, 1999–2014. MMWR Morb Mortal Wkly Rep 2018;67:845–849 30091969 10.15585/mmwr.mm6731a1 PMC6089335

[5] Ko JY, Patrick SW, Tong VT, Patel R, Lind JN, Barfield WD Incidence of Neonatal Abstinence Syndrome—28 States, 1999–2013. MMWR Morb Mortal Wkly Rep 2016;65:799–802 27513154 10.15585/mmwr.mm6531a2

[6] Conradt E, Flannery T, Aschner JL, et al. Prenatal opioid exposure: Neurodevelopmental consequences and future research priorities. Pediatrics 2019;144:e20190128 31462446 10.1542/peds.2019-0128 PMC6759228

[7] Azuine RE, Ji Y, Chang HY, et al. Prenatal risk factors and perinatal and postnatal outcomes associated with maternal opioid exposure in an urban, low-income, multiethnic US population. JAMA Network Open 2019;2:e196405 31251378 10.1001/jamanetworkopen.2019.6405 PMC6604084

[8] Creanga AA, Sabel JC, Ko JY, et al. Maternal drug use and its effect on neonates: A population-based study in Washington State. Obstet Gynecol 2012;119:924–933 22525903 10.1097/AOG.0b013e31824ea276

[9] Maeda A, Bateman BT, Clancy CR, Creanga AA, Leffert LR Opioid abuse and dependence during pregnancy: Temporal trends and obstetrical outcomes. Anesthesiology 2014;121:1158–1165 25405293 10.1097/ALN.0000000000000472

[10] National Institute of Drug Abuse. Indiana Opioid Summary. 2019. Available at: https://www.drugabuse.gov/drugs-abuse/opioids/opioid-summaries-by-state/indiana-opioid-summary Accessed 326, 2020

[11] Clark EAS, Esplin S, Torres L, et al. Prevention of recurrent preterm birth: Role of the neonatal follow-up program. Matern Child Health J 2014;18:858–863 23817726 10.1007/s10995-013-1311-0 PMC3823687

[12] Leaverton A, Lopes V, Vohr B, Dailey T, Phipps MG, Allen RH Postpartum contraception needs of women with preterm infants in the neonatal intensive care unit. J Perinatol 2016;36:186–189 26658122 10.1038/jp.2015.174

[13] Trussell J, Aiken ARA, Micks E, Guthrie K Efficacy, safety, and personal considerations. In: Hatcher RA, Nelson AL, Trussell J, et al., eds. Contraceptive technology, 21st ed. New York, NY: Ayer Company Publishers, Inc, 2018

[14] World Health Organization/Department of Reproductive Health and Research (WHO/RHR); Johns Hopkins Bloomberg School of Public Health (JHSPH)/Center for Communication Programs (CCP). Family planning: A global handbook for providers. Baltimore (MD): CCP: WHO, 2007

[15] Harris PA, Taylor R, Thielke R, Payne J, Gonzalez N, Conde JG Research electronic data capture (REDCap)—A metadata-driven methodology and workflow process for providing translational research informatics support. J Biomed Inform 2009;42:377–381 18929686 10.1016/j.jbi.2008.08.010 PMC2700030

[16] Harris PA, Taylor R, Minor BL, et al. The REDCap consortium: Building an international community of software platform partners. J Biomed Inform 2019;95:103208 31078660 10.1016/j.jbi.2019.103208 PMC7254481

[17] Centers for Disease Control and Prevention. Pregnancy Risk Assessment Monitoring System (PRAMS). 2016. Available at: https://www.cdc.gov/prams/pdf/questionnaire/Phase-8-Topics-Reference_508tagged.pdf Accessed 88, 2018

[18] National Institute of Drug Abuse. NIDA Drug Screening Tool. Available at: https://www.drugabuse.gov/nmassist/step/0 Accessed 926, 2018

[19] Desai RJ, Hernandez-Diaz S, Bateman BT, Huybrechts KF Increase in prescription opioid use during pregnancy among Medicaid-enrolled women. Obstet Gynecol 2014;123:997–1002 24785852 10.1097/AOG.0000000000000208 PMC4020039

[20] Alan Guttmacher Institute. 2009 National Survey of Reproductive and Contraceptive Knowledge. Available at: https://www.guttmacher.org/population-center/dataset/2009-national-survey-reproductive-and-contraceptive-knowledge Accessed 104, 2018

[21] Geist C, Aiken ARA, Sanders JN, et al. Beyond intent: Exploring the association of contraceptive choice with questions about Pregnancy Attitudes, Timing and How important is pregnancy prevention (PATH) questions. Contraception 2019;99:22–26 30125559 10.1016/j.contraception.2018.08.014 PMC6289803

[22] Terplan M, Hand DJ, Hutchinson M, Salisbury-Afshar E, Heil SH Contraceptive use and method choice among women with opioid and other substance use disorders: A systematic review. Prev Med 2015;80:23–31 25900803 10.1016/j.ypmed.2015.04.008 PMC4842019

[23] Kavanaugh ML, Jerman J Contraceptive method use in the United States: Trends and characteristics between 2008, 2012 and 2014. Contraception 2018;97:14–21 29038071 10.1016/j.contraception.2017.10.003 PMC5959010

[24] Kotha A, Chen BA, Lewis L, Dunn S, Himes KP, Krans EE Prenatal intent and postpartum receipt of long-acting reversible contraception among women receiving medication-assisted treatment for opioid use disorder. Contraception 2019;99:36–41 30114393 10.1016/j.contraception.2018.08.008 PMC6289834

[25] Krans EE, Kim JY, James AE, Kelley DK, Jarlenski M Postpartum contraceptive use and interpregnancy interval among women with opioid use disorder. Drug Alcohol Depend 2018;185:207–213 29462768 10.1016/j.drugalcdep.2017.12.023 PMC5889719

[26] Howell EM, Chasnoff IJ Perinatal substance abuse treatment: Findings from focus groups with clients and providers. J Subst Abuse Treat 1999;17:139–148 10435262 10.1016/s0740-5472(98)00069-5

[27] Matusiewicz AK, Melbostad HS, Heil SH Knowledge of and concerns about long-acting reversible contraception among women in medication-assisted treatment for opioid use disorder. Contraception 2017;96:365–369 28778423 10.1016/j.contraception.2017.07.167 PMC5643244

[28] Bornstein M, Gipson JD, Bleck R, Sridhar A, Berger A Perceptions of pregnancy and contraceptive use: An in-depth study of women in Los Angeles Methadone Clinics. Womens Health Issues 2019;29:176–181 30446331 10.1016/j.whi.2018.10.004 PMC6424631

[29] Bernard C, Wan L, Peipert JF, Madden T Comparison of an additional early visit to routine postpartum care on initiation of long-acting reversible contraception: A randomized trial. Contraception 2018;98:223–227 29778586 10.1016/j.contraception.2018.05.010 PMC6129199

[30] Daniels K, Jill D, Jones J Current contraceptive status among women aged 15–44: United States, 2011–2013. NCHS Data Brief 2014;173:1–8 25500343

[31] MacDorman MF, Eugene D, Thoma ME Trends in maternal mortality by sociodemographic characteristics and cause of death in 27 states and the District of Columbia. Obstet Gynecol 2017;129:811–818 28383383 10.1097/AOG.0000000000001968 PMC5400697

[32] Matthews TJ, MacDorman MF, Thoma ME Infant mortality statistics from the 2013 period linked birth/infant death data set. Natl Vital Stat Rep 2015;64:1–30 26270610

[33] Zerden ML, Tang JH, Stuart GS, Norton DR, Verbiest SB, Brody S Barriers to receiving long-acting reversible contraception in the postpartum period. Women's Health Issues 2015;25:616–621 26212318 10.1016/j.whi.2015.06.004

[34] Black KI, Haber PS, Lintzeris N Offering incentives to drug-using women to take up contraception: The ethical and clinical issues. Addiction 2012;107:1361–1362 22563688 10.1111/j.1360-0443.2012.03873.x

[35] Black KI, Day CA Improving access to long-acting contraceptive methods and reducing unplanned pregnancy among women with substance use disorders. Subst Abuse 2016;10:27–33 27199563 10.4137/SART.S34555 PMC4869602

[36] Dehlendorf C, Rodriguez MI, Levy K, Borrero S, Steinauer J Disparities in family planning. Am J Obstet Gynecol 2010;202:214–220 20207237 10.1016/j.ajog.2009.08.022 PMC2835625

[37] Borrero S, Schwarz EB, Creinin M, Ibrahim S The impact of race and ethnicity on receipt of family planning services in the United States. J Womens Health (Larchmt) 2009;18:91–96 19072728 10.1089/jwh.2008.0976 PMC2743980

[38] Dude A, Matulich M, Estevez S, Liu LY, Yee LM Disparities in postpartum contraceptive counseling and provision among mothers of preterm infants. J Womens Health (Larchmt) 2018;27:676–683 29359987 10.1089/jwh.2017.6561 PMC5962326

[39] Biggs MA, Rocca CH, Brindis CD, Hirsch H, Grossman D Did increasing use of highly effective contraception contribute to declining abortions in Iowa? Contraception 2015;91:167–173 25465890 10.1016/j.contraception.2014.10.009

[40] Goldthwaite LM, Duca L, Johnson RK, Ostendorf D, Sheeder J Adverse birth outcomes in Colorado: Assessing the impact of a statewide initiative to prevent unintended pregnancy. Am J Public Health 2015;105:e60–e66 10.2105/AJPH.2015.302711 PMC4539832 26180990

[41] Peipert JF, Madden T, Allsworth JE, Secura GM Preventing unintended pregnancies by providing no-cost contraception. Obstet Gynecol 2012;120:1291–1297 23168752 10.1097/aog.0b013e318273eb56 PMC4000282

[42] Krans EE, Cochran G, Bogen DL Caring for opioid-dependent pregnant women: Prenatal and postpartum care considerations. Clin Obstet Gynecol 2015;58:370–379 25775440 10.1097/GRF.0000000000000098 PMC4607033

